# Supplementary material for: Effects of Cinnamon Powder on Glucose Metabolism in Diabetic Mice and the Molecular Mechanisms
Source: Foods. 2023 Oct 20;12(20):3852. doi: 10.3390/foods12203852 (PMC10606646; doi:10.3390/foods12203852)
Supplement: Supplementary file 1 [file foods-12-03852-s001.zip › foods-2637026-supplementary.pdf]

Table S1. The composition of CP

Composition	Content
Total carbohydrates	14.20±1.10 g/100g dry weight
Protein content	4.71±0.08 g/100g dry weight
Total fat content	0.90±0.00 g/100g dry weight
Total dietary fiber	69.90±1.20 g/100g dry weight
Ash content	4.30±0.00 g/100g dry weight
Total polyphenols	71.62 ± 0.40μg/g dry weight
Total flavonoids	58.05 ±1.43 μg/g dry weight

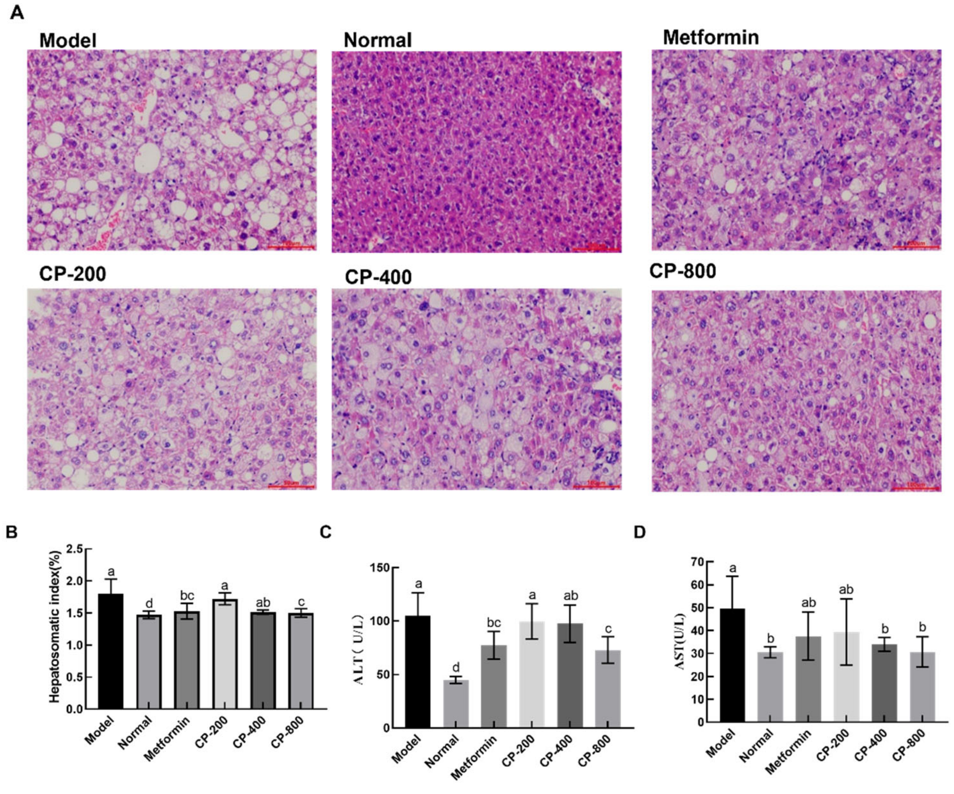

Figure S1. Effect of CP treatment on liver morphology (A) (H&E staining 200×), Hepatosomatic index values (B), ALT activity (C), and AST activity (D) in the liver tissues of T2DM mice.
